# Does digital government promote collaborative innovation? Evidence from the e-government pilot policy in China

**DOI:** 10.1371/journal.pone.0334131

**Published:** 2025-10-13

**Authors:** Jian Zhang, Yijie Li, Naiquan Liu, Jiansheng You

**Affiliations:** 1 School of Economics and Management, Tongii University, Shanghai, China; 2 School of public administration and policy, Shanghai University of Finance and Economics, Shanghai, China; Mazandaran University of Medical Sciences, IRAN, ISLAMIC REPUBLIC OF

## Abstract

In the context of the digital economy, digital government construction is increasingly regarded as a vital approach to enhancing urban innovation governance. This study uses panel data from 292 prefecture-level and above cities in China spanning 2012–2023 and adopts a difference-in-differences (DID) model centered around the 2017 e-government pilot policy to systematically evaluate the impact of digital government on intra-city collaborative innovation. The findings reveal that: (1) the e-government policy significantly increased the frequency and scale of patent collaborations within cities, indicating its positive effect in stimulating multi-actor cooperation, optimizing factor allocation, and facilitating knowledge sharing; (2) mechanism analysis shows that digital government enhances fiscal efficiency and administrative transparency, strengthens targeted support for science and education sectors, and promotes the spatial agglomeration of innovation resources toward urban cores, thereby reducing collaboration costs and improving the efficiency of innovation networks; (3) heterogeneity analysis indicates that the policy effect is more pronounced in highly market-oriented regions and non-provincial capital cities, reflecting differences in institutional adaptability and resource-driven incentives. The study provides micro-level empirical evidence for understanding the collaborative innovation effects of digital government and offers theoretical insights for regional governance and policy design.

## 1 Introduction

Against the backdrop of a new wave of technological revolution and industrial transformation, digitalization is profoundly reshaping production methods, organizational structures, and governance logic at an unprecedented pace [1]. The integration of technologies such as artificial intelligence, big data, cloud computing, and blockchain is accelerating the transformation of traditional government models toward “data-driven governance,” making digital government a key instrument globally for enhancing governance capacity and promoting high-quality development [2,3]. For developing economies, leveraging digital tools to break down administrative barriers, optimize resource allocation, and stimulate innovation vitality has become a central challenge for achieving inclusive growth and regional coordination [4,5]. In recent years, China has vigorously advanced e-government reforms, most notably the launch of a comprehensive e-government pilot policy in 2017, which marked a shift from fragmented service provision to platform-based coordination in digital governance [6,7]. The policy aims to build an efficient, transparent, and collaborative digital governance system through the integration of administrative platforms, promotion of data sharing, and optimization of service processes [8]. Its purpose extends beyond improving administrative efficiency, aiming also to reshape institutional environments and provide stronger support for urban innovation activities [9]. Nevertheless, whether digital government can indeed enhance collaborative innovation capacity in local cities through institutional optimization and data empowerment remains to be empirically verified. As a core component of regional innovation ecosystems, collaborative innovation emphasizes non-market-based cooperation among diverse actors based on trust and knowledge sharing, and its efficiency of collaborative innovation largely hinges on the stability of institutional environments, the fluidity of information, and the alignment of resource allocation [10,11]. Amid rapid urbanization and spatial fragmentation, how to strengthen the connectivity and collaborative efficiency among local firms, universities, and research institutions through digital governance platforms has become a crucial question for achieving regional innovation coordination [12,13]. Existing literature predominantly focuses on the impact of digital government on macro-level governance capacity and innovation outputs, while relatively few studies examine the mechanisms through which it affects collaborative innovation—especially due to the lack of micro-level data and quasi-experimental conditions for causal identification.

Although prior studies have yielded insights into how digital government fosters macro-level innovation output and improves government-business relations, quantitative evidence focusing specifically on “intra-urban collaborative innovation” remains insufficient. On the one hand, most existing research relies on macro-level indicators to capture e-government development, which limits the ability to assess its micro-level impact on innovation network structures and interactive mechanisms [14]. On the other hand, considerable disparities exist across cities in terms of digital governance capabilities, fiscal expenditure intensity, and information infrastructure, resulting in heterogeneous policy transmission pathways and innovation outcomes [15,16].

To address these gaps, this study takes the 2017 e-government pilot policy as a quasi-natural experiment and constructs a difference-in-differences (DID) model based on panel data from 292 prefecture-level and above cities in China from 2012 to 2023 to systematically evaluate the impact of digital government on intra-city collaborative innovation. Specifically, we develop a micro-level collaborative innovation index based on patent co-application behavior, integrating business registration data and geographic information to capture real technological cooperation among multiple local firms. The study also employs a series of robustness checks and mechanism identification analyses to trace the pathways through which digital government fosters local collaborative innovation.

This paper makes three primary contributions:

Innovative indicator construction: By using patent cooperation networks to build a micro-level indicator of intra-city collaborative innovation, this study captures actual technological collaborations between two or more local firms, overcoming the limitations of conventional innovation output indicators that fail to reflect inter-firm cooperation.

Multidimensional methodological advancement: In addition to the baseline DID model, we adopt parallel trend tests, placebo tests, propensity score matching DID (PSM-DID), spatial adjacency matching, and DDML techniques to ensure robust identification.

In-depth mechanism and heterogeneity analysis: The study investigates the mechanisms through which digital government promotes collaborative innovation from the perspectives of economic agglomeration, government spending in science and education, and spatial distribution of innovation actors. It also explores heterogeneous policy effects across cities with different levels of marketization and administrative hierarchy, providing empirical support for tailored governance strategies in digital government implementation.

Through these analyses, this paper not only offers micro-level evidence of the institutional performance of China’s digital governance capabilities but also provides practical insights for promoting balanced regional innovation and building a digitally empowered China.

## 2 Literature review

In recent years, with the rapid advancement of digital technologies and the ongoing transformation of governance paradigms, digital government has emerged as a crucial driver for the modernization of governance systems. It has evolved from “e-government” focused on informatization to “data-driven governance,” becoming a key institutional force in enhancing administrative efficiency and promoting high-quality economic development [17,18]. Existing studies on digital government have primarily emphasized its role in improving governance performance, optimizing public service delivery, and strengthening interactions between governments and enterprises [19,20]. On one hand, the construction of information systems and data platforms has significantly improved the accessibility and responsiveness of public services, thereby reshaping the operational logic of government organizations [21]. On the other hand, digital governance helps break down bureaucratic silos and hierarchical constraints, enabling governments to coordinate resources across departments more effectively and laying the institutional foundation for building a service-oriented state [22].

Amid the rise of innovation-driven economies, collaborative innovation has become a vital pathway to enhance the dynamism of regional innovation systems. It entails the joint creation of knowledge and reorganization of resources through non-market mechanisms involving multiple actors [23,24]. Prior research suggests that such collaboration not only relies on technological complementarity and inter-organizational trust but is also highly dependent on the stability and coherence of the external institutional environment [11,25]. In the context of urban governance, governments serve as platform-based intermediaries by providing fiscal support and public services, and more importantly, by formulating policies and institutional rules that shape the structure of innovation networks, thereby influencing collaborative efficiency and cooperative patterns [26,27]. Thus, understanding the formation logic of collaborative innovation requires a multi-dimensional perspective encompassing organizational relationships, spatial structures, and institutional supply.

Although existing literature has explored various mechanisms through which digital government affects urban innovation performance, research specifically focused on the dimension of “collaborative innovation” remains limited. From the institutional perspective, digital government enhances information transparency and reduces institutional transaction costs, facilitating smoother channels for inter-actor information sharing and policy alignment [28,29]. From the resource allocation perspective, it improves the government’s ability to allocate fiscal resources to knowledge-intensive sectors such as science and education, thereby providing stronger financial support for collaborative innovation [22,30]. From the spatial perspective, digital platforms concentrate resources and information in urban cores, accelerating the agglomeration of innovation factors within cities, reducing the physical and interactional costs among collaborators, and providing a favorable spatial foundation for knowledge spillovers and joint R&D activities [31,32]. Collectively, these mechanisms form the institutional logic chain through which digital government promotes collaborative innovation and supports the construction of more efficient and synergistic innovation ecosystems within cities.

While prior studies have preliminarily explored the impact of digital government on urban innovation, three key limitations remain: First, most research has focused on macro-level governance capacities or firm-level innovation outputs, with limited attention to “collaborative innovation” as an independent outcome, thus overlooking the interdependencies among multiple actors. Second, few studies have utilized micro-level data to construct patent collaboration networks, and in-depth analyses of how digital government influences cooperative relationships and interaction patterns among urban innovators remain lacking. Third, the mechanism through which digital government affects collaborative innovation is still unclear, and little systematic evidence exists on its heterogeneous effects across cities, leaving theoretical frameworks and empirical support underdeveloped.

In light of these gaps, this study centers on China’s e-government pilot policy as a case of digital government construction and provides new empirical evidence by evaluating its impact on micro-level urban innovation. More precisely, we treat the comprehensive e-government pilot launched in 2017 as a quasi-natural experiment and construct a city–government–patent three-dimensional panel dataset to assess the institutional incentives of digital government in the context of collaborative innovation. Relying on data from the China National Intellectual Property Administration and the National Enterprise Registration Database, we identify cooperative relationships among urban innovation actors and develop indicators of intra-city collaborative innovation based on the frequency and volume of patent co-applications. Finally, we employ a DID model, mechanism identification, and heterogeneity analysis to examine the policy’s incentive effects under varying levels of marketization and urban characteristics.

## 3 Theory analysis

The realization of intra-city collaborative innovation fundamentally relies on effective information sharing, efficient resource allocation, and institutional coordination among multiple actors. Under traditional governance systems, information silos, redundant administrative processes, and lagging services not only weaken governmental responsiveness but also raise implicit collaboration costs among innovation entities, thereby constraining the efficient integration and transformation of local innovation resources [33,34]. Particularly under conditions of increasing complexity in the distribution of innovation agents and diversification of collaboration pathways, failure to coordinate governance mechanisms efficiently can severely undermine the performance of regional innovation networks. As a core component of digital government, e-government promotes administrative process reengineering, interdepartmental data integration, and unified data platform development, significantly improving administrative efficiency and information transparency [35,36]. On one hand, this helps reduce institutional transaction costs for innovation actors to access policies, funding, and services, allowing for more accurate and efficient resource flow [37]. On the other hand, it strengthens data connectivity and interaction across departments, hierarchical levels, and organizations, thereby providing smoother institutional channels for intra-city knowledge sharing, resource integration, and technological collaboration [36]. The transformation of governance modes brought about by digital government not only optimizes the innovation environment, but also prompts local governments to play a more prominent role in platform-based service delivery and coordinated allocation, thus creating institutional support and informational momentum for collaborative innovation among enterprises, universities, and research institutions. This, in turn, enhances the breadth and depth of collaboration, and facilitates the evolution of regional innovation systems toward intensification and networkization [38]. Based on the theoretical analysis, this study proposes Hypothesis 1:

H1: E-government policies significantly enhance intra-city collaborative innovation.

Urban spatial structure plays a crucial supporting role in facilitating collaborative innovation. A compact urban form enhances the accessibility and frequency of interaction among innovation actors, reduces the costs of knowledge flow and collaborative R&D, and thereby increases the efficiency and dynamism of intra-city collaboration [39]. In contrast, a polycentric or dispersed spatial configuration often leads to factor mismatches and communication barriers, hindering the development of collaborative innovation [40]. As an institutional tool, e-government policy simplifies administrative procedures, improves service efficiency and information transparency, enhances the attractiveness of urban core areas to innovation resources, and accelerates the flow of talent, capital, and technology toward these areas—thus fostering a shift from polycentric to monocentric urban structures [41]. At the same time, digital governance platforms, by promoting information sharing and administrative integration, provide innovation entities with a more stable and intensive collaborative environment. The high-density interactions associated with urban agglomeration promote frequent knowledge exchange, strengthen the stability and resilience of collaborative networks, and further improve organizational efficiency within urban collaborative innovation systems [42]. Based on the above, this study proposes Hypothesis 2:

H2: E-government policies enhance intra-city collaborative innovation by improving urban spatial agglomeration.

Science and education resources are critical to sustaining urban innovation systems. Government fiscal investment in these areas not only reflects the level of commitment to building an innovation-friendly environment but also directly affects institutional capacity for talent development, research support, and knowledge dissemination [43]. In traditional fiscal systems, information asymmetries and inefficient allocation often lead to fragmented investments and structural imbalances, weakening the support base for innovation collaboration [44]. The advancement of e-government can effectively improve transparency and administrative efficiency, strengthen the government’s ability to identify innovation demands and educational needs, and enhance the precision of fiscal allocations. This approach ensures more funding can be directed toward knowledge-intensive areas such as R&D and talent cultivation, thereby providing a more stable resource foundation and institutional guarantee for collaborative innovation [45]. Moreover, concentrated fiscal resources also stimulate mechanisms such as platform construction, project funding, and high-level talent recruitment, enhancing organizational efficiency in urban innovation systems and further motivating multi-actor collaboration [46]. Based on the above, this study proposes Hypothesis 3:

H3: E-government policies promote intra-city collaborative innovation by increasing local government investment in science and education.

The spatial organization of innovation activities directly impacts the frequency of interaction and costs of collaboration among entities. Highly agglomerated spatial configurations can improve innovation efficiency, while dispersed structures tend to lead to resource mismatches and informational frictions that inhibit collaboration [47,48]. E-government, as a key element of digital governance, improves government service efficiency, streamlines administrative procedures, and strengthens information disclosure, thereby enhancing communication between innovation actors and government agencies and reducing institutional transaction costs [49]. Moreover, urban core areas—with their superior administrative services, information resources, and infrastructure—gradually strengthen their ability to attract talent, technology, and capital, driving innovation actors to concentrate in these regions. This agglomeration effect not only reduces physical distances but also improves collaboration efficiency and accelerates knowledge diffusion, facilitating high-frequency interactions and the rapid transformation of innovation outputs [50]. Innovation activities thus tend to cluster around city centers, reducing knowledge spillover barriers and collaborative R&D transaction costs, and strengthening the cohesion and dynamism of urban innovation networks. Therefore, by fostering spatial agglomeration of innovation activities, e-government creates a more efficient, lower-cost, and sustainable environment for collaborative innovation [51,52]. Based on the above, this study proposes Hypothesis 4:

H4: E-government policies promote intra-city collaborative innovation by enhancing the spatial agglomeration of innovation activities.

## 4 Research design

### 4.1 Model

#### 4.1.1 Baseline regression model.

To identify the impact of the e-government pilot policy on intra-city collaborative innovation, this paper constructs a baseline DID model:


$$\begin{array}{c} {co\_patent}_{it}=\beta_{\text{0}}+\beta_{\text{1}}{treat}_{i}\times {post\text{17}}_{t}+\sum \lambda X+\varepsilon_{i}+\varepsilon_{t}+\epsilon_{it} \end{array}$$ (1)

In the formula: *i* denotes the city and *t* denotes the time period. ${co\_patent}_{it}$ is the dependent variable, representing intra-city collaborative innovation. ${treat}_{i}$ is the treatment variable in the DID model, and ${post\text{1}\text{7}}_{t}$ is the dummy variable indicating the policy implementation period. X represents a series of control variables. $\varepsilon_{i}$ captures city fixed effects, $\varepsilon_{t}$ captures time fixed effects, and $\epsilon_{it}$ is the error term.

Prior to conducting regression analysis, this study first performs tests for homoskedasticity. The presence of heteroskedasticity among variables would compromise the reliability of estimation results. We estimate Equation (1) using OLS regression without controlling for fixed effects to specifically test for heteroskedasticity. The results demonstrate that for both collaboration frequency and joint patent count, the p-values of White tests are statistically significant (p < 0.05), indicating the presence of heteroskedasticity issues.

In the estimation process, the study clusters standard errors at the city level. This is because the same city may be subject to identical yet unobservable structural factors across different time periods, leading to potential correlations in error terms within the city. By clustering standard errors at the city level, the model allows for arbitrary forms of correlation and heteroskedasticity among residuals within the same city, while still maintaining the assumption of independence across different cities. This specification aligns with mainstream research practices.

This study does not involve human participants or animals. All data are obtained from publicly available statistical yearbooks and government databases. Therefore, approval from an institutional review board or ethics committee was not required.

#### 4.1.2 Event study method.

To validate the appropriateness of the DID model specification, this study adopts the event study method to test the parallel trend assumption before and after the policy implementation [53]. By constructing a series of time dummy variables, the researchers examine the trend of collaborative innovation levels between the treatment and control groups across each year surrounding the policy implementation, in order to determine whether any significant differences existed prior to the policy intervention.


$${co\_patent}_{it}=\beta_{\text{0}}+\sum_{k=\text{2012}}^{k=\text{2022}}\beta_{k}{treat}_{i}\times {I\left\{year=k\right\}}_{t}+\sum \lambda X+\varepsilon_{i}+\varepsilon_{t}+\epsilon_{it}$$ (2)

In the equation, *co_patent*_*it*_ represents the level of collaborative innovation in city *i* in year *t*, which is measured by both the frequency of cooperative innovation and the number of jointly owned patents. *treat*_*i*_ is a treatment group dummy variable: if city *i* is a pilot city for the e-government policy, then *treat*_*i*_${I\left\{year=k\right\}}_{t}$ denotes an indicator function that equals 1 when the condition is met (i.e., when the year is *k*), and 0 otherwise. Each year is interacted with the treatment group to construct interaction terms, and *β*_*k*_ estimates the change in collaborative innovation in the treatment group relative to the base year. In addition, the model includes city fixed effects *ε*_*i*_ and time fixed effects *ε*_*t*_, along with a set of control variables *X* to mitigate potential bias from omitted variables.

### 4.2 Variable

#### 4.2.1 Dependent variables.

Following the approach of Hanley et al. [54], this study identifies intra-city collaborative innovation behavior using patent-level data. Specifically, patents with more than one applicant, as recorded in the CNIPA (China National Intellectual Property Administration) database, are classified as collaborative patents, thereby capturing innovation activities involving actual cooperation among multiple entities. To accurately determine the spatial affiliation of collaborating entities, the study matches patent applicant information with China’s industrial and commercial enterprise registration data. Based on the registered address of applicants or enterprises, geographic coordinates are extracted and matched with the administrative boundaries of prefecture-level cities to identify the city location of each cooperating entity. If two or more applicants are located in the same city, the corresponding patent is defined as a sample of intra-city collaborative innovation.

Based on this collaborative patent data, two indicators are constructed to measure the level of intra-city collaborative innovation: (1) Collaboration Frequency (*Frequency*Number): The total number of patents jointly applied for by two or more applicants within a city, indicating the city’s capacity for co-created knowledge. To smooth the data distribution and reduce the influence of extreme values, both variables are log-transformed after adding one.

#### 4.2.2 Key explanatory variable.

This study uses the temporal variation in the implementation of the comprehensive e-government pilot policy across cities as a proxy for digital government development. Specifically, the core explanatory variable is constructed as the interaction between a city-level dummy variable and a time dummy variable. The city dummy takes a value of 1 if the city is located in a pilot province implementing the e-government initiative, and 0 otherwise. The time dummy is equal to 1 for years from 2018 onwards (the post-policy period), and 0 for earlier years.

#### 4.2.3 Control variables.

Following the methodological frameworks of Sotomayor et al. [55], Saeed et al. [56], and Shang et al. [57], the model includes a set of city-level control variables to account for other potential factors influencing intra-city collaborative innovation, thereby enhancing the robustness of the estimations. Specifically, the control variables include: Population Size (ln_*pop*): The natural logarithm of the total population of a city, reflecting the available labor force and overall scale of innovation participants. Per Capita GDP (ln_*pergdp*): The natural logarithm of per capita GDP, used to capture the level of regional economic development and its capacity to support innovation resources. Fiscal Expenditure (ln_*gov*): The logarithm of the local government’s general public budget expenditure, serving as a proxy for the degree of government intervention and fiscal support, which indirectly reflects its guidance and assurance role in innovation activities. Industrial Structure (*indu*): Measured by the proportion of the secondary industry’s added value to the regional GDP, indicating the level of industrialization. A higher share may imply stronger manufacturing capacity and a greater need for technological collaboration. Infrastructure Level (*road*): Represented by the natural logarithm of urban road area, serving as a proxy for transportation infrastructure. Better road accessibility facilitates inter-firm connections and knowledge spillovers. Table 1 summarizes the definitions of the variables.

**Table 1:** Descriptive of control variables.

Variable	Meaning	Descriptive
ln_pop	Population	Log of Total Urban Population
ln_pergdp	GDP per capita	Log of Regional GDP per Capita
ln_gov	Fiscal expenditure	Log of Local Government General Public Budget Expenditure
indu	Industry	Share of Secondary Industry
road	Infrastructure	Log of Urban Road Area

#### 4.2.4 Data sources.

This study utilizes panel data from 292 prefecture-level and above cities in China, spanning the period from 2012 to 2023, thereby ensuring comprehensive temporal coverage before and after the implementation of the e-government policy. Data on collaborative innovation are obtained from the China National Intellectual Property Administration (*CNIPA*), while firm-level information is drawn from the National Enterprise Registration Database. Control variables are primarily sourced from the ***China City Statistical Yearbook*** (2013–2024). Table 2 presents the descriptive statistics of the variables. Prior to conducting regression analysis, we examined multicollinearity among the variables and identified moderate but not severe multicollinearity in the model, which would not significantly affect the results. Additionally, we tested for potential serial correlation and found that all *p*-values were below 0.05, indicating the presence of this issue. Therefore, in the robustness checks section, we introduced the second-order lag term of the dependent variable into the model to mitigate the interference caused by serial correlation.

**Table 2:** Descriptive statistics.

Variable	Mean	SD	Min	p50	Max	N	VIF
Frequency	0.604	0.713	0.000	0.328	4.485	3460	–
Number	0.342	0.460	0.000	0.151	3.254	3460	–
treat	0.209	0.407	0.000	0.000	1.000	4068	1.12
post	0.583	0.493	0.000	1.000	1.000	4656	1.30
ln_pop	4.710	0.862	−2.303	4.667	7.841	3460	5.84
ln_pergdp	11.05	0.547	8.327	11.05	15.68	3407	2.61
ln_gov	14.02	1.060	10.10	13.94	18.38	3477	5.98
indu	0.441	0.118	0.0882	0.443	0.880	3473	1.38
road	7.221	1.005	1.609	7.152	10.39	3325	5.06

## 5 Results

### 5.1 Benchmark results

Table 3 presents the baseline regression results regarding the impact of the e-government policy on intra-city collaborative innovation. Columns (1) and (4) report the basic models that include only the core explanatory variable. Columns (2) and (5) extend the analysis by incorporating city and year fixed effects, while Columns (3) and (6) further control for city-level covariates.

**Table 3:** Benchmark results.

	(1)	(2)	(3)	(4)	(5)	(6)
	Frequency	Frequency	Frequency	Number	Number	Number
treat	0.473^***^			0.282^***^		
	(0.093)			(0.059)		
post	0.328^***^			0.184^***^		
	(0.023)			(0.016)		
treat × post	0.136^***^	0.136^***^	0.133^***^	0.124^***^	0.123^***^	0.121^***^
	(0.046)	(0.045)	(0.049)	(0.034)	(0.033)	(0.035)
N	3434	3434	3213	3434	3434	3213
adj. *R*^2^	0.172	0.817	0.825	0.162	0.828	0.830
Variable	N	N	Y	N	N	Y
City FE	N	Y	Y	N	Y	Y
Year FE	N	Y	Y	N	Y	Y

Note: ^*^, ^**^, ^***^ represent significance levels at 10%, 5%, 1%, respectively, with cluster stand errors in parentheses.

Across all model specifications, the coefficient of the policy interaction term (*treat × post*) remains positive and statistically significant at the 1% level, suggesting that the e-government pilot policy significantly promotes intra-city collaborative innovation. Specifically, the coefficient of the policy variable in Column (3) is 0.133, and in Column (6) is 0.121, indicating that—after accounting for city-level heterogeneity and temporal trends—the frequency of intra-city collaborative patent applications and the number of jointly owned patents increased by approximately 13.3% and 12.1%, respectively, in pilot cities post-policy implementation. These results support the hypothesized mechanism that “digital government initiatives enhance the coordination efficiency of local innovation factors by optimizing information flow and resource allocation systems.” On one hand, the establishment of e-government platforms improves administrative service efficiency and data accessibility, thereby reducing institutional transaction costs. On the other hand, following policy implementation, the efficiency of matching innovation factors across cities improves, enabling local firms to engage more actively in multi-agent and multi-stage collaborative innovation processes. Consequently, the vibrancy of intra-city knowledge co-creation is enhanced, thereby validating Hypothesis 1.

### 5.2 Robust test

#### 5.2.1 Parallel trend test.

**Fig 1:**
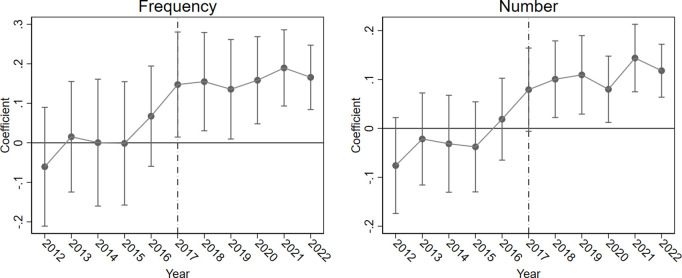
Parallel trend.

The parallel trend assumption is a critical prerequisite for causal identification in the DID approach. To test the validity of this assumption, this study adopts an event study methodology to analyze the dynamic changes in collaborative innovation before and after the implementation of the e-government policy [58]. The results are illustrated in Fig 1. As shown in Panel (a), prior to the implementation of the e-government policy from 2012 to 2016, the estimated coefficients of collaborative innovation frequency for the treatment group relative to the control group are statistically insignificant and exhibit minimal fluctuation. The results suggest that both groups followed a similar trend in collaborative innovation before the policy intervention, thereby supporting the parallel trend assumption. Panel (b) further confirms that during the same pre-policy period (2012–2016), none of the estimated coefficients for intra-city jointly owned patent counts reach statistical significance, indicating no systematic differences between the treatment and control groups. From a dynamic perspective, after the launch of the e-government pilot program in 2017, the estimated policy effect coefficients for both indicators show an upward trajectory and reach statistical significance starting from 2018. Specifically, the frequency of collaboration significantly increases in the year following the policy’s implementation and continues to rise thereafter. Meanwhile, the number of jointly filed patents begins to show steady growth from 2019 and maintains a robust positive impact after 2020.

#### 5.2.2 Placebo test.

**Fig 2:**
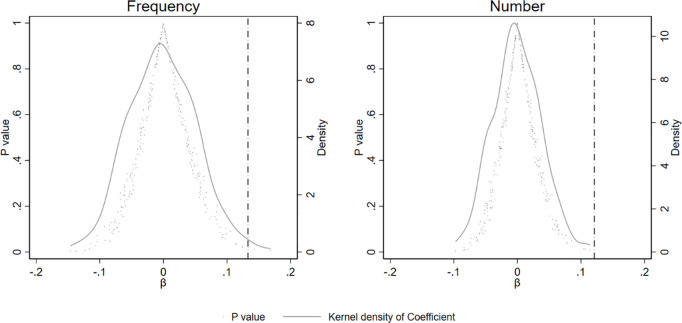
Placebo test.

To further verify the robustness of the core findings and eliminate potential interference from unobservable random factors that may have influenced the implementation of the e-government policy, this study conducts a placebo test based on a DID framework. The purpose of this test is to determine whether the identified policy effect arises from a genuine causal relationship rather than from incidental factors or specific sample characteristics. Specifically, drawing on the methodology of Li et al. [59] and Cantoni et al. [60], this paper constructs placebo treatment groups by randomly assigning “pseudo-policy cities” while maintaining the original distribution structure of cities. For each actual policy implementation year, a set of cities equal in number to the true pilot cities is randomly selected as the pseudo-treatment group, and a corresponding pseudo-policy indicator variable is created. This placebo variable is then substituted for the actual treatment variable in the DID regression model. The process is repeated 300 times to obtain distributions of placebo effect estimates and their associated *p*-values. The results are illustrated in Fig 2. By plotting the kernel density of the estimated coefficients, it is observed that the majority of the placebo effects are centered around zero and exhibit a symmetric distribution, which sharply contrasts with the significant deviation observed in the real estimates. This finding suggests that the positive impact of the e-government policy is not driven by random shocks, thereby further supporting the robustness of the study’s conclusions.

On the other hand, this study also conducts a placebo test from the perspectives of total patents and sole patents. While we argue that e-government policies have a positive effect on collaborative innovation, a potential concern is that such policies may simply boost overall innovation, with collaborative innovation being merely a byproduct. To address this, we examine total patents and sole patents at the city level. In contrast to co-owned patents, sole patents refer to those with only one applicant, meaning the patent is exclusively owned by that individual or entity. Based on this, we calculate per capita patent counts (*All*) and per capita sole patent counts (Solo) for each city and re-estimate the model. The results, presented in Table 4, show that e-government policies not only suppress overall innovation but also sole innovation. Therefore, the observed impact on innovation primarily stems from collaborative innovation.

**Table 4:** Placebo test.

	(1)	(2)	(3)	(4)
	All	All	Solo	Solo
treat × post	−0.152^***^	−0.122^**^	−0.147^***^	−0.117^**^
	(0.054)	(0.051)	(0.055)	(0.052)
N	3434	3213	3434	3213
adj. *R*^2^	0.909	0.932	0.907	0.929
Variable	N	Y	N	Y
City FE	Y	Y	Y	Y
Year FE	Y	Y	Y	Y

Note: ^*^, ^**^, ^***^ represent significance levels at 10%, 5%, 1%, respectively, with cluster stand errors in parentheses.

#### 5.2.3 PSM-DID.

To address potential endogeneity arising from sample selection bias, this study employs a PSM combined with DID approach to conduct robustness checks on the policy effect. Propensity scores are estimated using a binary choice model, and comparable samples are constructed using radius matching with calipers set at 0.05 and 0.01, respectively. After matching, all standardized differences in covariates fall below 10%, indicating a good quality of matching. The regression results are reported in Table 5, which show that the e-government policy consistently and significantly promotes urban collaborative innovation across different matching specifications, thereby validating the robustness of the main findings.

**Table 5:** PSM-DID.

	(1)	(2)	(3)	(4)
	Frequency	Frequency	Number	Number
Panel A: Caliper = 0.05				
treat × post	0.145^***^	0.139^***^	0.125^***^	0.124^***^
	(0.048)	(0.049)	(0.035)	(0.035)
N	3205	3205	3205	3205
adj. *R*^2^	0.816	0.818	0.818	0.822
Panel B: Caliper = 0.01				
treat × post	0.145^***^	0.138^***^	0.125^***^	0.122^***^
	(0.048)	(0.049)	(0.036)	(0.035)
N	3195	3195	3195	3195
adj. *R*^2^	0.813	0.816	0.815	0.818
Variable	N	Y	N	Y
City FE	Y	Y	Y	Y
Year FE	Y	Y	Y	Y

Note: ^*^, ^**^, ^***^ represent significance levels at 10%, 5%, 1%, respectively, with cluster stand errors in parentheses.

#### 5.2.4 Spatially adjacent city.

In the robustness check based on spatial proximity, this study further adopts a spatial matching strategy by restricting the control group to cities that are geographically adjacent to the treated cities. This approach aims to mitigate systematic biases caused by unobserved factors such as regional development disparities, differences in resource endowments, or variations in local policy environments. Such a setting enhances the net identification of policy effects and improves the credibility of causal inference. As shown in Table 6, when using the frequency of collaborative innovation and the number of jointly filed patents as dependent variables, the estimated coefficients of the interaction term *treat × post* remain significantly positive at the 5% and 1% levels, respectively. This indicates that, even after controlling for regional spatial characteristics, the e-government policy continues to have a significantly positive impact on urban collaborative innovation. Specifically, following the policy implementation, the collaboration frequency increased by approximately 0.191, and the number of co-applied patents rose by about 0.158. These results suggest that institutional coordination and information exchange mechanisms among cities were strengthened through the implementation of the policy pilot.

**Table 6:** Spatially adjacent city.

	(1)	(2)	(3)	(4)
	Frequency	Frequency	Number	Number
treat × post	0.192^***^	0.191^**^	0.159^***^	0.158^***^
	(0.071)	(0.077)	(0.049)	(0.051)
N	1363	1283	1363	1283
adj. *R*^2^	0.856	0.862	0.854	0.864
Variable	N	Y	N	Y
City FE	Y	Y	Y	Y
Year FE	Y	Y	Y	Y

Note: ^*^, ^**^, ^***^ represent significance levels at 10%, 5%, 1%, respectively, with cluster stand errors in parentheses.

#### 5.2.5 Double machine learning (DML).

Traditional OLS regression relies on strict assumptions regarding model specification and imposes high requirements on sample data, leading to several limitations. Chernozhukov et al. [61] proposed the DML framework, which addresses these issues through regularization for high-dimensional variable selection, orthogonalization to correct biases, and sample cross-fitting to avoid overfitting. Furthermore, machine learning methods relax constraints on the number of control variables and functional forms, thereby enabling more accurate estimation results [61,62]. Therefore, to effectively identify the causal impact of the e-government policy on collaborative innovation, this study adopts a partially linear model within the DML framework. A random forest algorithm is employed for prediction, with the sample split ratio set at 1:4. Additionally, the model incorporates quadratic terms of the control variables as well as province-by-year fixed effects to further account for unobserved heterogeneity. The corresponding estimation results are presented in Table 7.

**Table 7:** DDML test.

	(1)	(2)	(3)	(4)	(5)	(6)
Panel A: Frequency						
treat × post	0.907^***^	0.236^***^	0.243^***^	0.729^***^	0.128^***^	0.140^***^
	(0.040)	(0.035)	(0.036)	(0.047)	(0.050)	(0.050)
N	3434	3213	3213	3434	3213	3213
Panel B: Number						
treat × post	0.658^***^	0.167^***^	0.168^***^	0.524^***^	0.086^***^	0.090^***^
	(0.028)	(0.021)	(0.021)	(0.031)	(0.030)	(0.030)
N	3434	3213	3213	3434	3213	3213
Variable	N	Y	Y	N	N	Y
Variable^2	N	N	Y	N	Y	Y
City FE	Y	Y	Y	Y	Y	Y
Year FE	Y	Y	Y	Y	Y	Y
Province ×Year FE	N	N	N	Y	Y	Y
DML	RF	RF	RF	RF	RF	RF

Note: ^*^, ^**^, ^***^ represent significance levels at 10%, 5%, 1%, respectively, with cluster stand errors in parentheses.

#### 5.2.6 Spatial spillover effect.

Although this study has verified the parallel trends assumption, indicating no significant differences between the treatment and control groups prior to the policy implementation, and has employed neighboring cities of the treatment group as the control group for robustness checks to minimize systematic differences, potential spatial spillover effects may still exist. Specifically, if control group cities are linked to the treatment group in some way, they might also be partially affected by the policy, leading to an underestimation of the treatment effect. To address this concern, this study incorporates spatial factors into the analysis to rule out such interference.

Specifically, we adopt spatial econometric methods, calculating inter-city distances based on geographic centroids to construct a spatial weight matrix (*W*). For the analysis, we employ a SDM model while controlling for time and city fixed effects. Additionally, since spatial econometrics requires balanced panel data, we first process the dataset, resulting in a loss of 177 observations (compared to Table 3). To ensure that sample trimming does not bias the results, we first re-estimate the model using OLS on the trimmed sample. As shown in Columns 1 and 2 of Table 8, the results remain nearly identical to those in Table 3, confirming that sample trimming does not affect our findings.

**Table 8:** Spatial spillover test.

	(1)	(2)	(3)	(4)
	OLS		Spatial Econometrics	
	Frequency	Number	Frequency	Number
treat × post	0.129^***^	0.121^***^	0.106^**^	0.103^***^
	(0.047)	(0.035)	(0.052)	(0.037)
W × treat × post			0.011	0.005
			(0.627)	(0.401)
rho			−1.402^***^	−1.601^***^
			(0.249)	(0.280)
N	3036	3036	3036	3036
adj. *R*^2^	0.833	0.837		
Variable	Y	Y	Y	Y
City FE	Y	Y	Y	Y
Year FE	Y	Y	Y	Y

Note: ^*^, ^**^, ^***^ represent significance levels at 10%, 5%, 1%, respectively, with cluster stand errors in parentheses.

Building on this, we introduce the spatial econometric model to analyze spatial spillover effects. The results, presented in Columns 3 and 4 of Table 8, reveal that after accounting for spatial spillovers, the estimated treatment effect decreases by approximately 14.9% to 17.8%. While the model confirms spatial dependence in the dependent variable, the key policy treatment effect remains statistically significant, rejecting the presence of substantial spatial spillover effects.

### 5.3 Mechanism test

#### 5.3.1 Agglomeration of economic activity.

Based on the LandScan Global Population Distribution Database and the China Urban Built-up Area Dataset (1992–2020) released by the National Tibetan Plateau Scientific Data Center [63], this study identifies the spatial extent of built-up areas for each prefecture-level and above city in China. The dataset provides delineated boundaries of urban built-up areas, which are defined as closed polygonal regions representing the economic footprint of each city. For each polygon, total population and raster-based land area are calculated, and their ratio yields the urban population density. Using this framework and combined with grid-level population data, we construct a population density indicator for each built-up area—measured as the ratio of total population to the built-up land area—to capture the degree of spatial agglomeration of economic activities within cities. Furthermore, following the methodology of Li and Liu [64], we compute the polycentricity and the number of centers in each city to characterize their spatial morphology. Empirical analysis reveals that the e-government policy significantly increases population density within built-up areas while reducing both the degree of urban polycentricity and the number of urban centers. The empirical results indicate that the e-government policy has played an important role in promoting the transformation of cities from polycentric to monocentric spatial structures, as reported in Table 9. Such “agglomeration effects” not only improve the internal allocation of resources and mobility of innovation factors, but also create a more favorable institutional and spatial environment for localized collaborative innovation—thereby enhancing both the breadth and efficiency of intra-city innovation collaboration, this finding confirms Hypothesis 2.

**Table 9:** Mechanism: Agglomeration of economic activity.

	(1)	(2)	(3)
	density	ploy	number_of_center
treat × post	0.026^*^	−0.051^***^	−0.693^***^
	(0.014)	(0.018)	(0.143)
N	3181	3181	3181
adj. *R*^2^	0.918	0.837	0.890
Variable	Y	Y	Y
City FE	Y	Y	Y
Year FE	Y	Y	Y

Note: ^*^, ^**^, ^***^ represent significance levels at 10%, 5%, 1%, respectively, with cluster stand errors in parentheses.

#### 5.3.2 Government spending on technology and education.

From the perspective of government resource allocation and innovation incentives, this section further examines the impact of the e-government policy on municipal investment in science and education. We use government spending on technology and education as indicators of investment in these domains, and assess the policy effects both in terms of total expenditures and per capita values. The mechanism analysis reveals that the e-government initiative significantly increased local government fiscal investment in science and education. As shown in Table 10, following the policy implementation, the coefficients for both total science expenditure (*tech*) and per capita science expenditure (*tech_pop*), as well as for total education expenditure (*edu*) and per capita education expenditure (*edu_pop*), are all positive and statistically significant at the 1% level. This suggests that the e-government policy effectively expanded the scale of investment while enhancing per capita allocation efficiency. Mechanistically, the e-government reform enhanced the flexibility and precision of fiscal coordination by simplifying procedures and improving efficiency. On one hand, the policy enabled governments to better identify cities’ actual demands for science and education funding, improving targeting accuracy. On the other hand, the digital governance platform strengthened interdepartmental coordination and information sharing, facilitating smoother flows of talent, capital, and technology. Furthermore, the increase in science and education spending not only occurred in absolute terms but also reflected a structural shift toward knowledge-intensive sectors. These developments created a more favorable institutional and resource environment for collaboration among local innovation actors, thereby enhancing both the capacity and efficiency of urban collaborative innovation. Hypothesis 3 is thus confirmed.

**Table 10:** Mechanism: Government spending on technology and education.

	(1)	(2)	(3)	(4)
	tech	tech_pop	edu	edu_pop
treat × post	0.144^***^	0.143^***^	0.296^***^	0.282^***^
	(0.055)	(0.049)	(0.107)	(0.069)
N	3211	3211	3212	3212
adj. *R*^2^	0.869	0.782	0.922	0.884
Variable	Y	Y	Y	Y
City FE	Y	Y	Y	Y
Year FE	Y	Y	Y	Y

Note: ^*^, ^**^, ^***^ represent significance levels at 10%, 5%, 1%, respectively, with cluster stand errors in parentheses.

#### 5.3.3 Agglomeration of innovation.

Based on the method proposed by Harari [31], this study identifies the spatial centers of patent activity within each city by using patent address data, and further calculates the average and maximum distances from each patent to the city’s innovation center. These indicators are used to evaluate the spatial distribution of innovation activities within cities. In Table 11, columns (1) and (2) use the average and maximum patent-to-center distances as dependent variables, respectively. The results show that both indicators declined significantly after the implementation of the e-government policy, with significance levels at 5% and 1%, respectively. This indicates that the policy played a substantial role in facilitating the spatial concentration of innovation factors. From a mechanistic perspective, e-government enhanced governance efficiency and information symmetry, thereby creating favorable conditions for innovation resources to concentrate in urban core areas. On one hand, digital governance improved interactions between enterprises and the government, lowered the cost of accessing public policies and services, and increased the attractiveness of core areas to talent, technology, and capital. On the other hand, the agglomeration of innovation actors led to higher interaction frequency and collaboration efficiency, reducing the transaction costs of knowledge spillovers and joint R&D. Nevertheless, the observed agglomeration effect may be driven by multiple mechanisms rather than a single pathway. Beyond the reduction of transaction costs, agglomeration can also stem from resource reallocation dynamics, as talent and capital are more likely to move toward urban cores once administrative barriers are lowered. Moreover, institutional improvements under digital governance—such as greater transparency and clearer policy signals. These complementary explanations suggest that the spatial concentration of innovation activities is the outcome of intertwined economic, informational, and institutional forces. Hypothesis 4 is thus validated.

**Table 11:** Mechanism: Agglomeration of innovation.

	(1)	(2)
	mean_zx	max_zx
treat × post	−1.416^**^	−4.853^***^
	(0.644)	(1.611)
N	3213	3213
adj. *R*^2^	0.829	0.859
Variable	Y	Y
City FE	Y	Y
Year FE	Y	Y

Note: ^*^, ^**^, ^***^ represent significance levels at 10%, 5%, 1%, respectively, with cluster stand errors in parentheses.

### 5.4 Heterogeneity test

#### 5.4.1. Marketization degree.

The sample is divided into high-marketization and low-marketization cities based on the median level of marketization across all cities. Columns (1) and (2) of Table 12 present the effect of e-government on the frequency of collaborative innovation. The results show that in high-marketization cities, the e-government policy significantly increases the frequency of collaborative innovation (*p* < 0.01). In contrast, the coefficient for low-marketization cities is 0.113, which is positive but only weakly significant. Similarly, columns (3) and (4) indicate that the effect of e-government on the number of collaborative innovation entities is significantly stronger in high-marketization cities (*p* < 0.01) compared to low-marketization ones (*p* < 0.1), with the difference between the two groups being statistically supported (**p* *< 0.1).

**Table 12:** Heterogeneity: Market index.

	(1)	(2)	(3)	(4)
	Market High	Market Low	Market High	Market Low
	Frequency	Frequency	Number	Number
treat × post	0.212^***^	0.113	0.173^***^	0.102^*^
	(0.060)	(0.074)	(0.044)	(0.052)
N	1569	1575	1569	1575
adj. *R*^2^	0.825	0.799	0.835	0.790
P value	0.050		0.030	
Variable	Y	Y	Y	Y
City FE	Y	Y	Y	Y
Year FE	Y	Y	Y	Y

Note: ^*^, ^**^, ^***^ represent significance levels at 10%, 5%, 1%, respectively, with cluster stand errors in parentheses.

These findings suggest that cities with higher levels of marketization have advantages in institutional environments, property rights protection, and factor mobility, allowing the institutional dividends of e-government to be more effectively translated into collaborative innovation momentum. On one hand, high-marketization cities possess more developed market mechanisms and stronger capacities for allocating innovation resources, which help stimulate firms’ willingness and capacity to collaborate. On the other hand, digital governance platforms operate more efficiently in these cities, with shorter policy transmission chains, enabling faster aggregation and interaction of innovation elements. Moreover, to eliminate the influence of time-invariant city-specific characteristics and temporal trends, city fixed effects and year fixed effects are included in the regression model.

#### 5.4.2 Administrative hierarchy of cities.

Based on administrative hierarchy, the sample is divided into provincial capital cities and non-capital cities to examine differences in the policy’s impact on collaborative innovation. Columns (1) and (2) of Table 13 present the effects of e-government on the frequency of collaborative innovation. The results indicate that the policy effect is not statistically significant in provincial capitals (*p* > 0.1), while in non-capital cities, the effect is 0.151 and statistically significant at the 1% level. Similarly, columns (3) and (4) show that, regarding the number of collaborative innovation entities, the coefficient for non-capital cities is significantly positive (0.128, *p* < 0.01), whereas no significant effect is observed in provincial capitals. These findings suggest that the e-government policy has a more pronounced effect on promoting collaborative innovation in non-capital cities. The underlying reason is that, compared to provincial capitals, non-capital cities typically have more limited innovation resources and fewer external collaboration channels. Under traditional governance systems, these cities find it more difficult to attract inter-city cooperation or engage with high-quality external actors. E-government improves administrative efficiency and transparency, reduces collaboration barriers, and thereby strengthens connections among local innovation entities, fostering a more cohesive internal innovation network. Conversely, provincial capitals already possess strong capabilities in resource allocation and external connectivity, with innovation activities often extending beyond local boundaries and favoring inter-city or cross-regional collaboration, which limits the marginal impact of the e-government policy.

**Table 13:** Heterogeneity: Urban administrative level.

	(1)	(2)	(3)	(4)
	Capital	un Capital	Capital	un Capital
	Frequency	Frequency	Number	Number
treat × post	−0.082	0.151^***^	−0.002	0.128^***^
	(0.124)	(0.052)	(0.085)	(0.037)
N	329	2884	329	2884
adj. *R*^2^	0.913	0.780	0.913	0.790
*P* value	0.000		0.000	
Variable	Y	Y	Y	Y
City FE	Y	Y	Y	Y
Year FE	Y	Y	Y	Y

Note: ^*^, ^**^, ^***^ represent significance levels at 10%, 5%, 1%, respectively, with cluster stand errors in parentheses.

## 6. Conclusion and policy implications

### 6.1 Conclusion

Based on panel data from 292 prefecture-level and above cities in China spanning the years 2012–2023, this study takes the 2017 e-government pilot policy as a quasi-natural experiment and applies a DID model to systematically evaluate the impact of digital government construction on intra-city collaborative innovation. The main findings are as follows:

E-government policy significantly enhances intra-city collaborative patent activity, both in terms of frequency and volume. The conclusion holds under a series of robustness checks, indicating that digital government initiatives can effectively stimulate cooperation willingness among diverse local innovation actors. By improving the efficiency of resource matching and knowledge sharing, digital government serves as a critical policy tool to strengthen collaborative innovation capacity within cities.

Mechanism analysis reveals that e-government improves administrative processes and transparency, enhances the efficiency of fiscal resource allocation, and steers science and education expenditures toward knowledge-intensive sectors. While facilitating targeted resource deployment, e-government also strengthens the agglomeration of innovation factors, encourages the concentration of innovation entities in urban core areas, reduces the cost of collaboration, and boosts the operational efficiency of the local innovation ecosystem.

In terms of heterogeneity, the policy effect of e-government is more pronounced in highly market-oriented regions and non-provincial capital cities. The former demonstrates stronger institutional responsiveness, while the latter, operating under relatively limited resource endowments, exhibits greater potential for incentive-driven collaboration. The policy thus plays a crucial role in unlocking innovation potential and fostering convergence in regional innovation capabilities.

### 6.2 Policy implications

In line with implementation scholarship [65,66], we recommend a people-, process- and learning-oriented implementation package rather than a technology-only approach:

1. Workforce development and institutional capacity

Effective policy implementation requires not only digital infrastructure but also sustained investment in human capital. We recommend earmarking a fixed share of project budgets for continuous staff training, change-management workshops, and professional development. Beyond one-off programs, agencies should establish competency frameworks with annual upskilling targets to ensure personnel can adapt to evolving technologies. Building this institutional capacity reduces the risk of costly systems being underutilized due to insufficient expertise.

2. Inter-agency coordination and governance mechanisms

Technological systems can only function effectively when supported by clear governance arrangements. Agencies should formalize cooperation through memoranda of understanding (MOUs) and adopt management tools such as RACI charts to define roles in data sharing, platform operation, and dispute resolution. Appointing a lead coordinating agency with measurable key performance indicators (KPIs) ensures accountability and timeliness. Such mechanisms prevent fragmentation, enhance cross-departmental trust, and facilitate smoother policy implementation.

3. Performance metrics and adaptive learning

To avoid a purely input-oriented focus, performance must be evaluated through outcome-based metrics. In addition to traditional indicators like budget execution, governments should track reductions in processing time, rates of cross-departmental case closure, and tangible outputs of collaboration such as joint patents. These KPIs should be reviewed at regular intervals, with procedures adjusted according to results. This adaptive learning process creates a feedback loop that continuously improves efficiency and ensures resources are aligned with policy goals.

4. Risk Management and sustainability frameworks

Large-scale digital and institutional reforms are prone to risks such as staff turnover, vendor lock-in, or poor integration with legacy systems. Establishing a live risk register with named owners and clear mitigation timelines helps anticipate and address these challenges early. Periodic post-implementation reviews can capture lessons learned and feed them back into future projects. Moreover, ensuring data governance safeguards—including access control, classification standards, and audit trails—strengthens long-term sustainability and builds stakeholder confidence.

### 6.3 Discussion

A key contribution of this study is to provide micro-level causal evidence on how digital government construction fosters collaborative innovation within Chinese cities. By exploiting the 2017 e-government pilot policy as a quasi-natural experiment and constructing a novel patent co-application indicator, this paper demonstrates that digital government significantly increases both the frequency and intensity of intra-city collaborative innovation. Mechanism analyses further reveal that the policy enhances fiscal efficiency in science and education spending, promotes the spatial agglomeration of innovation factors, and strengthens the organizational capacity of urban innovation networks. These findings enrich the literature by linking digital governance reforms with the dynamics of collaborative innovation, offering new insights into how institutional modernization contributes to the evolution of urban innovation ecosystems. Nevertheless, several issues merit further discussion. First, while patent-based indicators provide objective and verifiable measures of technological collaboration, they may not fully capture the diversity of innovation activities. Informal cooperation, non-technological innovations, and industry-specific differences in patenting propensity are often excluded from patent statistics. Moreover, strategic patenting behavior may distort the extent to which co-application data reflect genuine knowledge sharing. Thus, our findings should be interpreted as reflecting patent-related collaborative innovation rather than the entirety of innovative activities within cities. Future research could triangulate patent data with alternative indicators such as joint publications, project-level collaboration records, or survey-based measures to provide a more comprehensive understanding.
